# Supplementary material for: Unmet Healthcare Needs of Children in Vulnerable Families in South Korea: Finding from the Community Child Center Child Panel Survey
Source: Int J Environ Res Public Health. 2020 Nov 7;17(21):8241. doi: 10.3390/ijerph17218241 (PMC7664643; doi:10.3390/ijerph17218241)
Supplement: Supplementary file 1 [file ijerph-17-08241-s001.pdf]

Table 1. Differences between two groups of children based on diseases in the previous year.

(*N* = 515)

Characteristics		Category	Group without disease (*n* = 175)	Group with disease (*n* = 340)	χ^2or t	*p*
Predisposing factors	Gender	Boys	91(52.0)	146(42.9)	3.82	0.06
		Girls	84(48.0)	194(57.1)		
	Area of residence	Metropolitan region	55(31.4)	128(37.6)	1.95	0.17
		Non-metropolitan region	120(68.6)	212(62.4)		
Enabling factors	Type of health insurance	National Health Insurance	76(43.4)	149(43.8)	0.01	1.00
		Medical Aid	99(56.6)	191(56.2)		
	Living with parents	Both parents	94(53.7)	224(65.9)	7.24	0.01
		One parent/other	81(46.3)	116(34.1)		
	Caregiver after school	Present	106(60.6)	167(49.1)	6.09	0.02
		Absent (one day or more/week)	69(39.4)	173(50.9)		
	Degree of conversation with parents		2.0±0.9	2.1±0.9	-1.32	0.19
	Degree of activities with parents		2.6±1.1	2.8±1.1	-2.34	0.02
	Degree of indifference by parents		2.2±0.8	2.1±0.8	1.09	0.28
	Degree of abuse by parents		2.5±1.0	2.6±1.0	-1.77	0.08
	Duration of daily stay at CCC (min)		184.1±82.5	174.9±75.1	1.26	0.21
	Satisfaction with teachers at CCC		3.8±0.8	3.7±0.9	0.87	0.39
Need factors	Number of diseases		0.0±0.0	1.6±1.0	-21.00	<.001
	Perceived physical symptoms		1.7 ±0.5	1.9±0.6	4.16	<.001
